# Individual Molecular Dynamics of an Entangled Polyethylene Melt Undergoing Steady Shear Flow: Steady-State and Transient Dynamics

**DOI:** 10.3390/polym11030476

**Published:** 2019-03-12

**Authors:** Mohammad Hadi Nafar Sefiddashti, Brian J. Edwards, Bamin Khomami

**Affiliations:** Materials Research and Innovation Laboratory (MRAIL), Department of Chemical and Biomolecular Engineering, University of Tennessee, Knoxville, TN 37996, USA; mnafarse@vols.utk.edu

**Keywords:** entangled polymer melts, linear polymers, nonequilibrium molecular dynamics simulations, steady and startup shear flows

## Abstract

The startup and steady shear flow properties of an entangled, monodisperse polyethylene liquid (C_1000_H_2002_) were investigated via virtual experimentation using nonequilibrium molecular dynamics. The simulations revealed a multifaceted dynamical response of the liquid to the imposed flow field in which entanglement loss leading to individual molecular rotation plays a dominant role in dictating the bulk rheological response at intermediate and high shear rates. Under steady shear conditions, four regimes of flow behavior were evident. In the linear viscoelastic regime ($\dot{\gamma }<\tau_{d}^{-1}$), orientation of the reptation tube network dictates the rheological response. Within the second regime ($\tau_{d}^{-1}<\dot{\gamma }<\tau_{R}^{-1}$), the tube segments begin to stretch mildly and the molecular entanglement network begins to relax as flow strength increases; however, the dominant relaxation mechanism in this region remains the orientation of the tube segments. In the third regime ($\tau_{R}^{-1}<\dot{\gamma }<\tau_{e}^{-1}$), molecular disentangling accelerates and tube stretching dominates the response. Additionally, the rotation of molecules become a significant source of the overall dynamic response. In the fourth regime ($\dot{\gamma }>\tau_{e}^{-1}$), the entanglement network deteriorates such that some molecules become almost completely unraveled, and molecular tumbling becomes the dominant relaxation mechanism. The comparison of transient shear viscosity, $\eta^{+}$, with the dynamic responses of key variables of the tube model, including the tube segmental orientation, S, and tube stretch, λ, revealed that the stress overshoot and undershoot in steady shear flow of entangled liquids are essentially originated and dynamically controlled by the $S_{xy}$ component of the tube orientation tensor, rather than the tube stretch, over a wide range of flow strengths.

## 1. Introduction

The study of flow properties of polymeric solutions and melts has a rich history of perplexing the physicists and engineers who have endeavored to understand and model the many and varied physical responses of these complex fluids to an imposed flow field. In particular, the description of fast flows of macromolecular fluids has proven to be a difficult challenge. Although many continuum level theories have proven capable of describing gross rheological data in the linear and weakly nonlinear viscoelastic flow regimes (i.e., at low to intermediate values of the strain rate relative to a characteristic relaxation time of the fluid), most of these have not been able to provide a quantitative description of the flow properties of solutions and melts at high flow strength. There are many possible reasons one could cite to explain this state of dysfunction, but the overall reason is abundantly clear: for polymeric fluids experiencing strong flow conditions, all of the physical and dynamical phenomena occurring within these materials have not been understood and accounted for in the prevailing mathematical models.

Developing reliable mathematical models necessarily depends upon complementary experimentation. A debilitating feature of rheological experimentation, however, is that these seemingly simple experiments typically only provide bulk-scale measurements that have effectively been averaged over macroscopic length and time scales. As a consequence, any dynamic behavior that is of much shorter length and time scale than those of the measuring instrument are effectively washed out of the system response, even though they contribute to the overall response. Therefore, for much of the 20th century, rheologists had little in the way of small length and time scale information to guide attempts at improved mathematical modeling.

The 21st century is proving to be a golden age of rheological discovery. New experimental methods have been developed which are beginning to tap into small time and length scale phenomena that have a dramatic impact on the bulk rheological response of a polymeric liquid, particularly under conditions of strong flow. Furthermore, the present century has seen the rise of a new form of scientific exploration; i.e., *virtual experimentation*. Advances in computational algorithms and efficiency have led to a new paradigm in experimentation that, under the right circumstances, can lead to a powerful new means to probe the small length and time scale phenomena that dominate the bulk rheological responses of polymeric fluids under strong flow conditions.

The primary advantage of virtual experimentation of an atomistically detailed polymer chain over experiment is that every chain within the sample can be examined individually, not simply the bulk rheological or microstructural response. This allows much more detailed information to be gleaned from the simulation with respect to the experiment, as statistically meaningful correlations can be established via ensemble averaging of the dynamical behavior of each individual chain. Additionally, simulations are readily amenable to topological analysis, extending equilibrium properties such as tube diameter, primitive path length, and number of entanglements to nonequilibrium flow situations [1,2,3,4,5,6,7]. Certainly, bulk-averaged properties, such as the conformation and stress tensors, can still be calculated, but also with the ability to examine the effects of short timescale individual chain dynamics upon them. Ultimately, more and better information at the microscopic scale should lead to better rheological and microstructural models of polymeric liquids under flow.

Recent evidence collected via virtual experimentation of monodisperse atomistic melts has demonstrated that a flow-induced disentanglement of polymer macromolecules occurs at high strain rates in steady shearing flow. This reduction in interchain constraints leads to the onset of individual molecular retraction and rotation cycles, which occur within oriented tube-like structures composed of the highly-extended surrounding chain molecules. Eventually, the tube network disintegrates as the chains become effectively disentangled, allowing them to tumble with characteristic frequencies similarly to corresponding macromolecules in dilute solution. This new phenomenon has been observed via nonequilibrium molecular dynamics (NEMD) simulations of molten polyethylenes in the unentangled and moderately-entangled molecular-weight regimes (i.e., polyethylenes ranging up to C_700_H_1402_) [1,2,3,4,8,9,10,11,12,13,14,15]. This unexpected observation from atomistic simulations has already been hypothesized to explain some of the difficulties that manifest in flow models for high strain-rate flows [16,17,18].

In the present contribution, prior results of unentangled (liquids ranging in molecular weight roughly up to C_250_H_502_), mildly entangled (C_400_H_802_), and moderately entangled (C_700_H_1402_) polyethylene melts are extended to a highly entangled system, C_1000_H_2002_, thus completing the entire suite of virtual experiments of flexible, monodisperse linear macromolecular fluids ranging from unentangled alkane liquids to highly entangled polyethylene melts. Hence this publication presents the final piece of the puzzle to those that preceded it, providing a full description of the rich, complex dynamical behavior and the underlying physical mechanisms that give rise to it as macromolecular chain length increases from several carbon units up to 1000. Moreover, this work extends prior studies that were focused on steady-state dynamics to the transient response of these entangled liquids under startup of shear conditions. The data and analysis presented in the remainder of this article will help enable physicists and engineers to develop new and improved models for the bulk rheological behavior of these macromolecular fluids covering all the relevant length and time scales.

## 2. Simulation Methodology

Equilibrium and nonequilibrium molecular dynamics simulations of a monodisperse, linear, C_1000_H_2002_ melt were performed in the NVT ensemble at a constant density of 0.766 g/cm^3^ (corresponding to a pressure of 1 atm) and constant temperature of 450 K. Four different rectangular simulation cells were chosen for different shear rate ranges in order to minimize the computational cost by optimizing the simulation box size and number of particles. Table 1 summarizes the cell sizes in various directions as well as the number of particles and applicable Wi range. In the nonlinear viscoelastic regime (Wi>1), the box dimension in the flow direction (x) was larger than the dimensions in the gradient (y) and neutral (z) directions to ensure minimal system size effects at high shear rates where chains orient and stretch in the direction of flow. These dimensions were chosen based on the same considerations in terms of the chain end-to-end distance at different Wi which were employed for a shorter C_700_H_1402_ chain liquid in prior work [2]. The smallest simulation cell, containing 20,000 particles, was equilibrated for more than 8 times the longest relaxation (disengagement) time before any data were gathered for analysis. The simulation cells containing 40,000 and 60,000 particles were created by replicating the equilibrated small simulation cell respectively once and twice in the x-direction, then equilibrated for one disengagement time. The longest cell was created by replicating the equilibrated cell containing 60,000 particles, twice in the x-direction, and then equilibrated for 0.8 disengagement time. It should be mentioned that the transient data were obtained using only a single independent initial equilibrium configuration to minimize the computational cost; however, this configuration varied from one simulation cell size to another—see Table 1. Although ideally such data should be collected using more than one independent initial configuration at each Wi, based on prior experience, this has only a slight effect on the data presented in this work, considering the sufficiently large number of particles in the simulation cells.

The Siepmann–Karaboni–Smit (SKS) united-atom potential model [19] was used to quantify the energetic interactions between the atomistic components of the polyethylene liquid. This is the same potential model employed in many other prior simulation studies [1,2,3,8,10,11,12,13,14,17,18,20,21,22,23] to represent energetic interactions between either -CH_3_ for the end-groups of the chains or -CH_2_- groups for interior carbon atoms along the chain backbone. (Please refer to one of the references cited above for a detailed discussion of the SKS model equations and parameters.)

The NEMD equations of motion were used to perform the NEMD simulations, which were maintained at a constant temperature of 450 K using a Nosé–Hoover thermostat [24,25,26,27,28,29,30,31]. The set of evolution equations for the particle positions and momenta were integrated within the Large-scale Atomic/Molecular Massively Parallel Simulator (LAMMPS) environment, which is implemented using the p-SLLOD equations of motion [29,30,31]. (Note that for steady-state and startup shear flow as considered herein the SLLOD and p-SLLOD algorithms are the same.) Boundary conditions were periodic at all box surfaces with a deforming simulation cell in the x direction. The equations were integrated using the reversible-Reference System Propagator Algorithm (r-RESPA) [32] with two different time steps. The long time step was 4.70 fs, which was used for the slowly varying nonbonded Lennard-Jones interactions, and the short time step was 1.176 fs (one-fourth of the long time step) for the rapidly varying forces including bond-bending, bond-stretching, and bond-torsional interactions. The relaxation time of the thermostat was set equal to 100 times the long time step. These time steps are longer than those used in many of the prior studies [1,2,3,4,8,11,12,13,14,17,18,21,22]; however, a series of test simulations were performed at various *Wi* to ensure that the new (longer) time steps produced statically equivalent results as the prior (shorter) time steps. Furthermore, these time steps have been used successfully in recent NEMD studies of planar elongational flows of entangled polyethylene melts [20,23]. Without this modification of the time steps, the simulations reported in this article would have been computationally intractable.

A wide range of Weissenberg numbers was examined over the interval [0; 0.01, 11,700], corresponding to the quiescent system and shear rates within the range $2.2\times 10^{3}s^{-1}\leq \dot{\gamma }\leq 2.2\times 10^{9}s^{-1}$. The topological analysis was performed using the Z1 code developed by Kröger [5], which reduces atomistic configurations to a primitive path network in which the chains are not allowed to cross each other as the algorithm simultaneously minimizes the contour length of each polymer molecule [6]. This method uses geometrical methods rather than dynamical algorithms to minimize the contour lengths of primitive paths in the most computationally efficient manner. The code further defines positions of kinks along the 3-dimensional primitive path of each chain, which are assumed to be roughly proportional to the number of entanglements per chain. Results of the code can be used to interpret other important reptative parameters, such as the effective tube diameter and entanglement strand length. The Z1 code has been compared with other topological analysis techniques by Shanbhag and Kröger [7].

## 3. Results and Discussion

### 3.1. Quiescent Properties

Equilibrium properties of the system can be calculated from the simulation results and compared with the predictions of reptation theory. The ensemble average squared end-to-end distance, $\left\langle R^{2}\right\rangle$, and radius of gyration, $\left\langle R_{g}{}^{2}\right\rangle$, were calculated as 20,107 ${Å}^{2}$ and 3353 ${Å}^{2}$, respectively, directly from the equilibrium simulation data. The theoretical fully extended chain end-to-end distance, ${\left|R\right|}_{\max}$, for a C_1000_H_2002_ molecule is 1290.2 Å. These values may be used to approximate the Kuhn length as $=\langle R^{2}\rangle /{\left|R\right|}_{\max}=15.58\;Å$, and the number of Kuhn segments as $N=\langle R^{2}\rangle /b^{2}=82.79\approx 83$. Entanglement network properties were evaluated using the Z1-code [5]. Specifically, the average primitive chain contour length, ⟨L⟩=508.6 Å, was obtained based on this analysis. These basic properties of the entangled liquid can be used in conjunction with reptation theory to estimate other (theoretical) system properties. The ensemble average entanglement density is thereby estimated as

(1) $$Z=\frac{\left\langle L\right\rangle }{a\;}=\frac{Nb^{2}}{a^{2}}=\frac{{\left\langle L\right\rangle }^{2}}{\langle R^{2}\rangle \;},$$

where a is the tube diameter. From this expression, Z=12.9 and $a=\langle L\rangle /Z=\langle R^{2}\rangle {}^{1/2}/Z^{1/2}=39.5\;Å$. All of these values are in good agreement with the values estimated for C_400_H_802_ and C_700_H_1402_ molecules in prior work [2,3,18]. The entanglement molecular weight for polyethylene at 443 K of $M_{e}=1150$ g/mol was reported by Fetters, et al. [33], which can be used to estimate an experimental entanglement density of $Z=M/M_{e}=12.9$, and tube diameter $a=\langle R^{2}\rangle {}^{1/2}/Z^{1/2}=40.6\;Å$. These values are in excellent agreement with the simulation results.

The diffusivity of the liquid can be evaluated from the slope of the chain center-of-mass mean-squared displacement (MSD) versus time. According to its definition, $D_{G}$ is 1/6 of this slope at long times:

(2) $$D_{G}=\underset{t\to \infty }{\lim}\;\frac{1}{6t}{\left\langle (R_{G}\left(t+\tau \right)-R_{G}\left(\tau \right)\right)}^{2}\rangle ,$$

where $R_{G}\left(t\right)$ is the position of the chain center of mass at time t. Using this method, the diffusivity of C_1000_H_2002_ is calculated as $1.28\times 10^{-12}{\;m}^{2}/s$. Note that this value makes it possible to calculate a key model parameter of the theory, the friction coefficient, ξ [34],

(3) $$D_{G}=\frac{k_{B}T\;a^{2}}{3N^{2}\xi b^{2}},$$

where $k_{B}$ is the Boltzmann constant and T is the absolute temperature.

According to reptation theory, the Rouse and disengagement timescales are governed by the expressions [34]

(4) $$\tau_{R}=\frac{\xi N^{2}b^{2}}{3\pi^{2}k_{B}T\;},$$

(5) $$\tau_{d}=\frac{1}{\pi^{2}}\frac{\xi N^{3}b^{4}}{k_{B}T\;a^{2}}.$$

Substituting ξ from Equation (3) into Equation (4),$\;\tau_{d}$ can be expressed as a function of $D_{G}$:

(6) $$\tau_{d}=\frac{1}{3\pi^{2}}\frac{R^{2}}{D_{G}}.$$

Hence, the theoretical value of the disengagement time (i.e., according to the equations of reptation theory after $D_{G}$ has been estimated from the simulations) is calculated as 5305 ns. Note that from Equations (1), (4) and (5), the ratio $\tau_{d}/\tau_{R}=3Z$, which leads to a theoretical Rouse time of 137 ns.

The entanglement time is governed by the reptation-based Equation [3]

(7) $$\tau_{e}=\frac{\pi }{36}\frac{R^{2}}{D_{G}Z^{3}},$$

which gives a value of 6.4 ns. This is very close to the values calculated for the C_400_H_802_ and C_700_H_1402_ liquids (5.1 ns and 6.4 ns, respectively). These values are consistent with theoretical arguments suggesting that the entanglement time is independent of the molecular weight of the polymeric liquid.

**Figure 1:**
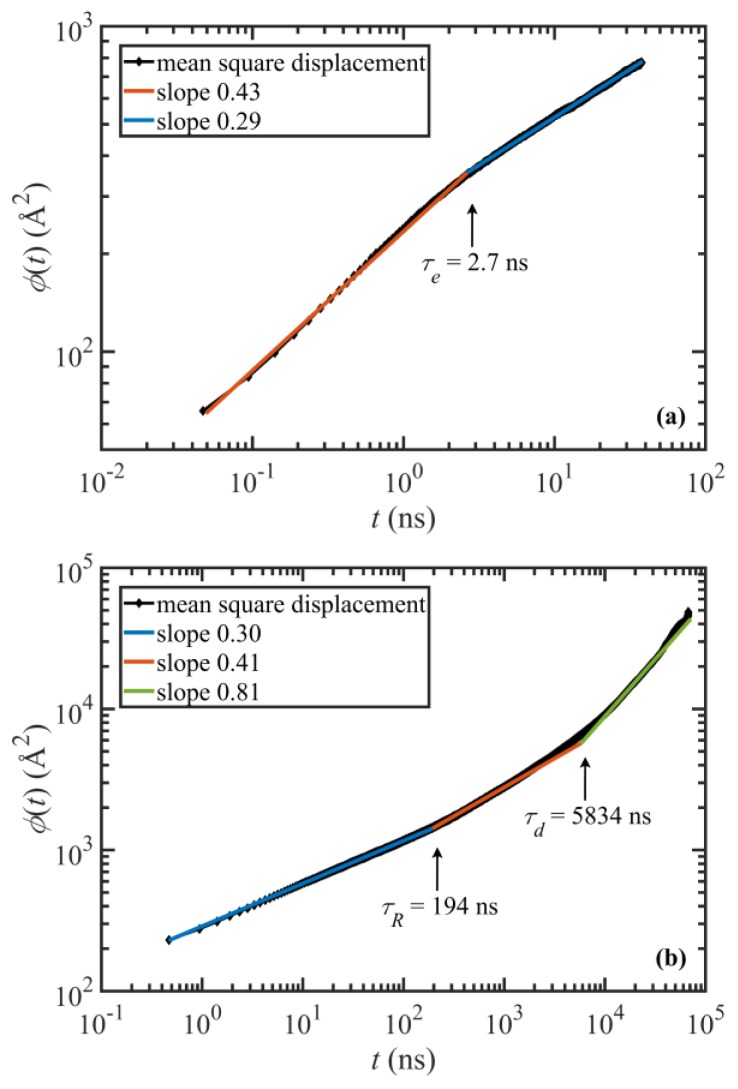
Short (**a**) and long (**b**) timescale segmental MSD versus time of the (500) centermost chain atomic units.

The characteristic relaxation times can also be estimated directly from the equilibrium simulation results using the characteristic breaks in the segmental mean-square displacement (MSD) plot versus time [34]. The segmental MSD is defined as $\phi \left(t\right)=\langle {\left(r_{n}\left(t+\tau \right)-r_{n}\left(\tau \right)\right)}^{2}\rangle$, where $r_{n}$ is the position vector of the n-th monomer (i.e., the n-th -CH2- unit). In order to minimize chain-end effects, only the 500 monomers in the middle of each chain were included in these calculations. The details of the calculations are explained in prior publications [2,3]. Figure 1 displays these plots for very short times (a) and long times (b). As shown in the figure, the disengagement, Rouse, and entanglement times turn out to be, respectively, 5834 ns, 194 ns, and 2.7 ns. Both the Rouse and disengagement times are mildly overpredicted as compared to the theoretical values. Also, the ratio $\tau_{d}/3\tau_{R}=10$, which is smaller than the expected theoretical value of *Z* = 12.9; this suggests that the Rouse time is significantly overpredicted by this method. On the other hand, $\tau_{e}$ is underpredicted compared to the theoretical value; however, it is in good agreement with the entanglement times calculated for the C_400_H_802_ and C_700_H_1402_ melts using the same method.

Another robust method for direct calculation of the disengagement time from the simulation data is to fit a sum of exponential functions to the autocorrelation function of the chain end-to-end unit vector, $\langle u_{i}\left(\tau \right)\cdot u_{i}\left(\tau +t)\right\rangle =\overset{p}{\underset{i=1}{\sum }}c_{i}\exp\left(-t/\tau_{i}\right)$, where the longest value of $\tau_{i}$ is considered as the disengagement time. p is the minimum number of exponential terms (5 in this case) that results in the best fit (i.e., the closest coefficient of determination, R-squared, to unity, using a nonlinear least-squares method), and $c_{i}$ are fitting constants of order unity. The disengagement time, based on this method, is calculated to be 5270 ns, which agrees very well with the theoretical prediction (5305 ns).

**Figure 2:**
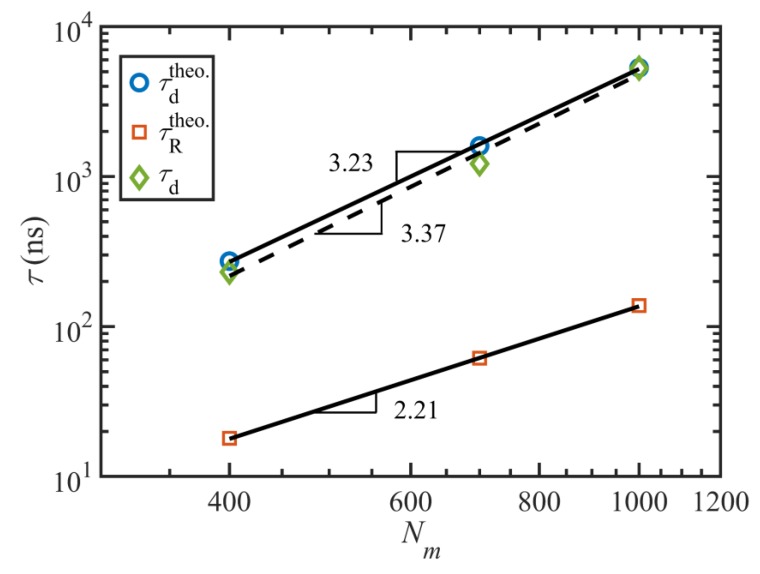
Characteristic timescales as functions of molecular length. Power-law exponents agree well with experimental observations.

Figure 2 displays a log-log plot of the Rouse and disengagement times versus chain length (number of monomers per chain), as well as their relevant power-law fitting and exponents. The data for the C_400_H_802_ and C_700_H_1402_ liquids were obtained from prior work [2,3]. These plots show that the power-law exponents for the disengagement time calculated from either the theoretical method or the fitting method are about 3.3±0.1, in good agreement with experimental measurements for entangled polymers. This suggests that all physical phenomena, including contour length fluctuations (CLF), constraint release (CR), and of course reptation, are captured well by the simulations under quiescent conditions. One may expect a power-law exponent of 3.0 for the theoretical values of the disengagement time based on reptation model predictions alone; however, it should be noted that although Equations (3)–(5) were used for calculation of the theoretical characteristic times, the diffusivity (or equivalently the friction coefficient) was calculated from the simulation results and consequently includes all important physical phenomena, as explained earlier. In fact, the power-law exponent for the diffusivity itself is −2.3, in excellent agreement with experimentally observed values [34,35]. The same analysis is valid for the power-law exponent of the theoretical Rouse time, which scales as $N_{m}^{2.2}$ rather than the theoretical exponent of 2, which again should be attributed to CLF and CR effects [2]. Table 2 summarizes the results of the calculations for the equilibrium characteristic relaxation times of the C_1000_H_2002_ melt obtained from the theoretical and MSD methods. In the rest of this chapter, we use the values $\tau_{d}=5270$ ns (exponential method), $\tau_{R}=137$ ns (theoretical method), and $\tau_{e}=6.4$ ns (theoretical method) for the characteristic time scales of the C_1000_H_2002_ liquid (unless otherwise noted).

### 3.2. Steady Shear Flow Properties

#### 3.2.1. Steady-State Structural and Topological Properties

The steady-state microstructural and topological properties of a C_1000_H_2002_ melt undergoing simple shear flow are qualitatively very similar to those of the C_400_H_802_ and C_700_H_1402_ liquids, which were discussed in detail in prior publications [2,3,4,18]. These results are presented concisely herein; interested readers can refer to the cited references for more comprehensive discussions. Overall, steady-state shear properties of the C_1000_H_2002_ melt exhibit four distinct regions of behavior ($\dot{\gamma }<\tau_{d}^{-1}$, $\tau_{d}^{-1}<\dot{\gamma }<\tau_{R}^{-1}$, $\tau_{R}^{-1}<\dot{\gamma }<\tau_{e}^{-1}$, $\tau_{e}^{-1}<\dot{\gamma }$), as noted previously for the C_700_H_1402_ liquid [2].

**Figure 3:**
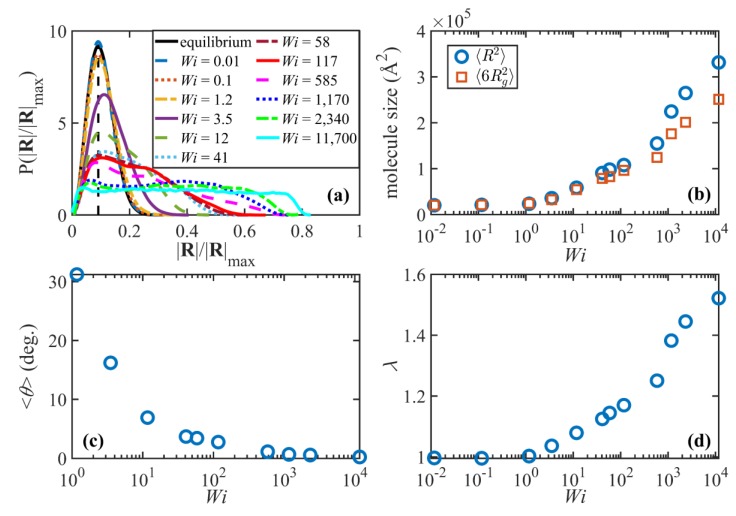
Probability distribution of the normalized magnitude of the end-to-end vector $\left|R\right|/{\left|R\right|}_{\max}$ at various values of Wi (**a**), the mean-square of the end-to-end vector and 6 times the radius of gyration vs. Wi (**b**), ensemble average chain orientation angle with respect to the flow direction vs Wi (**c**), and tube stretch vs Wi (**d**).

The probability distribution functions (PDFs) of the normalized end-to-end distance and the chain size (measured in terms of ensemble averages of chain end-to-end distance and six times the radius of gyration, respectively) are displayed for various values of Wi in Figure 3a,b. In the linear viscoelastic regime (Wi≤1), the PDFs are Gaussian and remain essentially unchanged from the quiescent state. The ensemble averages of the squared end-to-end distance and (6 times the) radius of gyration also remain constant and almost equal to each other in this regime. This suggests that the flow is too weak to significantly perturb the global molecular sizes. Keep in mind that the timescale of the flow is larger than the disengagement time (i.e., $\dot{\gamma }<\tau_{d}^{-1}$), implying that the constituent macromolecules have ample time for diffusive action to maintain their quiescent configurational properties even though the overall tube network begins to orient along a preferred direction in the shear plane relative to the direction of flow. Note that the ratio $\left\langle R^{2}\rangle /\langle R_{g}^{2}\right\rangle$ approaches the theoretical value of 6 for long flexible Gaussian chains.

Figure 3c displays the ensemble average orientation angle, ⟨θ⟩, as a function of Wi. ⟨θ⟩ is calculated as the angle between the principal eigenvector of the ensemble average of the unit end-to-end vector dyadic product, $\left\langle u_{i}u_{i}\right\rangle$, and the flow (x) direction. The orientation angle decreases from the zero-shear-rate limit of 45° (not shown in the figure) to about 30° at Wi=1. Finally, the tube stretch is shown as a function of Wi in Figure 3d. The tube stretch is defined as the ratio $\lambda =\langle L\rangle /L_{0}$, where $L_{0}$ is the quiescent primitive path length. Both ⟨L⟩ and $L_{0}$ are calculated using the Z1 code. No chain stretch is observed in the linear viscoelastic region, as expected.

As the flow enters the weakly nonlinear regime, $\tau_{d}^{-1}<\dot{\gamma }<\tau_{R}^{-1}$ (or equivalantly 1<Wi≤38), the orientation angle drops dramatically to values smaller than 5° and plateaus around 1–2° at higher Wi. The PDF of the end-to-end distance begins deviating from the equilibrium Gaussian distribution by developing a tail at higher values of $\left|R\right|/{\left|R\right|}_{\max}$, indicating that a portion of the macromolecules have become partially extended by the applied flow. Notably, the PDF peak is still approximately at the same location as the equilibrium distribution, which suggests that the overall conformation of a significant number of chains has not yet been perturbed. The growth of molecular size and the deviation from Gaussian behavior can also be inferred from Figure 3b, especially for Wi>10 where $\langle R^{2}\rangle \;$and $6\langle R_{g}^{2}\rangle$ begin to diverge. (Note that there is no theory which indicates these two quantities are equivalent under flow conditions.) Interestingly, the tube network also begins to extend moderately in in this shear-rate region (Figure 3d). This is an important observation because it contradicts the common notion of tube-based models that no stretching occurs for $\dot{\gamma }<\tau_{R}^{-1}$. Quantitatively, Figure 3d indicates that tubes are stretched about 16% at $\dot{\gamma }\sim \tau_{R}^{-1}$, which is not negligible although just a fraction of the maximum theoretical tube stretch, $\lambda_{\max}=2.77$.

**Figure 4:**
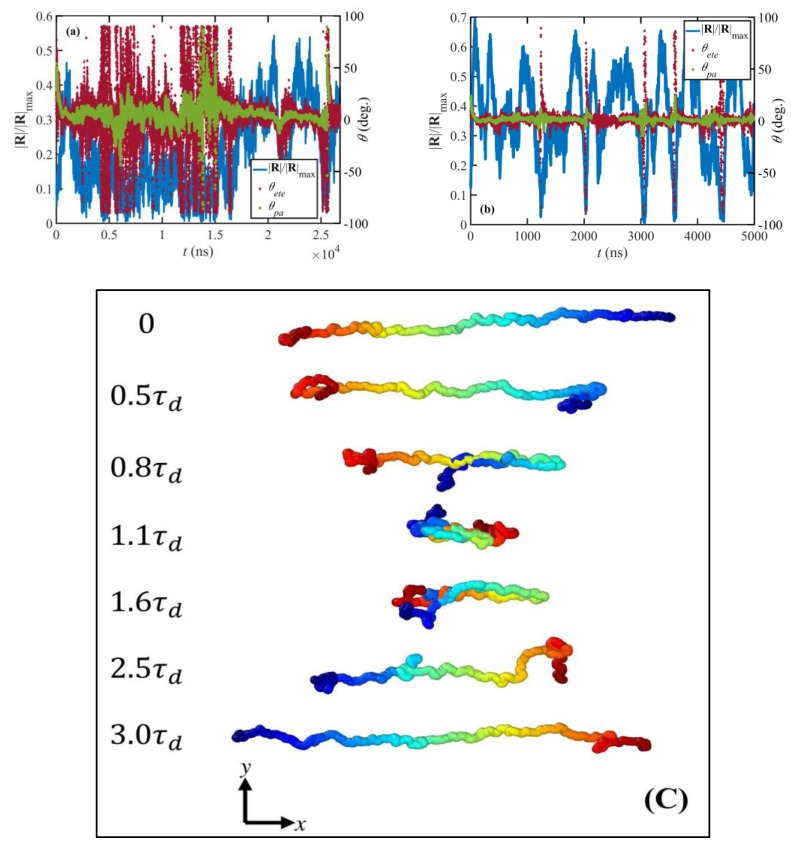
Normalized end-to-end vector and orientation angle vs. time for a random chain at Wi=58 (**a**) and Wi=1170 (**b**). A time progression of configurations of a random chain at Wi=117 is depicted in panel (**c**). $\theta_{ete}$ is the angle between the chain end-to-end vector and the flow direction (x) and $\theta_{pa}$ is the angle between the primary axis of the chain and the flow direction.

The third shear-rate regime of dynamical behavior is the range $\tau_{R}^{-1}<\dot{\gamma }<\tau_{e}^{-1}$ (approximately 40≤Wi<800). Within this region, vorticity excursions start playing an important role in the system properties. Brownian fluctuations caused by the vorticity of the shear field lead to random excursions of the chain ends outside of the confining tubes; some of these excursions, especially those with shear-plane projections that possess negative orientation angles relative to the flow direction, induce rotation and retraction quasi-periodic tumbling cycles of the individual molecules at moderate and high shear rates similar to those observed in previous work [2,3,8,14,18,22]. A typical cycle begins as a chain molecule stretches and aligns in the flow direction (see Figure 4c). At this point, due to the flow vorticity, chain ends fold backward along the spine of the molecule and slide toward the middle of the chain until the molecule collapses into a compressed configuration. Then the orientation of the chain flips as the chain ends cross and the molecule unravels until it adopts a stretched conformation again that concludes a half cycle. At the lower end of this range (Wi=40 to 100), the cycle is very irregular, almost chaotic. Here, the macromolecules will reside in the compressed state for a long period of time (see Figure 4a). Under this condition, the chain ends, which are typically very close to each other, exhibit a wagging behavior due to Brownian motion, passing each other back and forth multiple times before the molecule begins to reextend. As a consequence, the orientation angle of the chain end-to-end vector, $\theta_{ete}$, oscillates haphazardly between −90° and 90° as evident in Figure 4a. The orientation angle of the chain primary axis, $\theta_{pa}$, however, does not oscillate as much as $\theta_{ete}$ suggesting that the body of the molecule does not wag like a solid object. (The primary axis of the molecule is defined as the eigenvector corresponding to the largest eigenvalue of the molecule gyration tensor.) Yet, $\theta_{pa}$ changes rapidly between positive and negative values when the molecule is in a collapsed and highly compressed state, indicating that the coiled chains wag for some indefinite period of time before they begin to unravel.

In the uppper portion of the range $\tau_{R}^{-1}<\dot{\gamma }<\tau_{e}^{-1}$ (i.e., 100<Wi<800), the dynamical behavior of the macromolecules is much more regular and resembles the tumbling behavior observed in prior work [2,3,8,14,18,22]. During a typical cycle, the chain end-to-end distance varies dramatically from high values associated with the stretched configurations to values that are even smaller than the average equilibrium end-to-end distance. This is manifested in the wide non-Gaussian bimodal probability distribution function at this flow regime, as displayed in Figure 3a. Specifically, the peak at low values of $\left|R\right|/{\left|R\right|}_{\max}$ shifts to the left as Wi increases and occurs at extensions smaller than the equilibrium peak, indicating the increasing population of the collapsed configurations during the course of the tumbling cycle. At the same time, the ensemble average molecule size (Figure 3b) and tube stretch (Figure 3d) increase with Wi in this flow region. Based on theoretical arguments, this is the region wherein tube stretch becomes significant. As mentioned earlier, Figure 3d shows that tube stretching begins at lower flow strength than theoretically expected; however, tube stretch in the third flow region is apparently of a different nature than within the second flow regime. In the third region, λ scales as $\lambda^{0.04}$ while this power-law exponent is 0.03 in the second range, $\tau_{d}^{-1}<\dot{\gamma }<\tau_{R}^{-1}$. This suggests that the tube stretch is influenced by the tumbling dynamics of the individual macromolecules, and that it has an influential contribution to the shear stress and constitutes a major relaxation mechanism in this intermediate flow strength regime. Note that the time-average orientation angle of the molecules is very close to its plateau value in the third region and does not change significantly, indicating that the chain end-to-end vectors are almost completely aligned in the flow direction on the molecular length scale, although not necessarily on the tube segment length scale.

The fourth and final flow regime is the strong flow region where $\dot{\gamma }>\tau_{e}^{-1}$, approximately Wi>800. Although the molecules continue to stretch in this region (Figure 3b,d), the molecular size and the tube stretch ultimately attain plateau values, which are significantly smaller than their corresponding maximum theoretical values. The tube stretch profile has an inflection point around $\dot{\gamma }\sim \tau_{e}^{-1}$ where the curvature changes from positive to negative. This signals a new regime where the tube stretch becomes saturated as chain rotation becomes the more dominant dynamic mechanism. The shape of the end-to-end distance distribution curve is also very different in this high Wi region compared to that at lower Wi regimes. Specifically, the distributions become relatively flat with a characteristic rotational peak at low $\left|R\right|/\left|R_{\max}\right|$ and a stretch peak that emerges at very high Wi. (See Figure 3a. The stretch peaks can also be easily recognized in C_400_H_802_ and C_700_H_1402_ systems [18].) These flat distributions, which become wider as Wi increases, are attributed to the more regular molecular rotation cycles at very high shear rates, as discussed by Nafar Sefiddashti, et al. [3]. The skewed distributions within the intermediate Wi regime suggest that during a rotation cycle individual molecules spend on average a longer time at collapsed (or less stretched) configurations than they do at relatively stretched configurations [18], or that some of the chains have not yet stretched enough to begin their rotation cycles (see Figure 4a). Both cases lead to unbalanced lifetimes for various configurations, and consequently irregular rotation cycles. Within the high Wi regime, on the other hand, molecules undergo more regular periodic cycles. Hence various configurations between a highly stretched chain and a tightly packed coil have fairly similar lifetimes or probabilities (see Figure 4b), which manifest in the flat probability distribution of the end-to-end distance [18].

**Figure 5:**
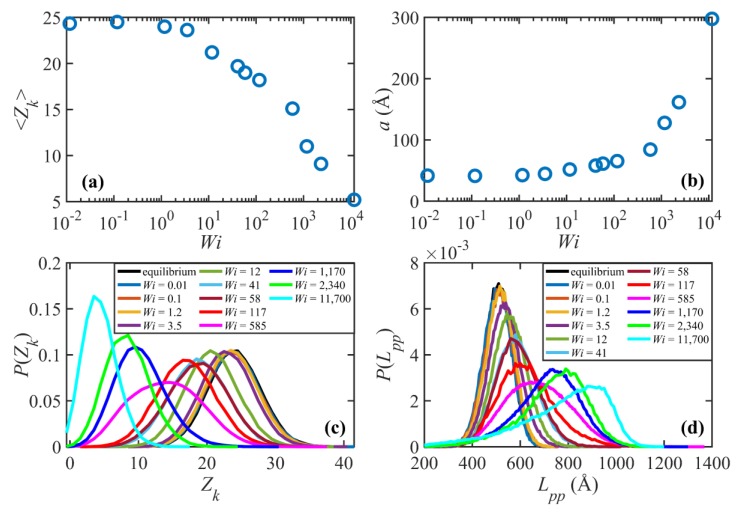
Average number of entanglements per chain versus Wi (**a**), the effective tube diameter versus Wi (**b**), and probability distributions of the number of entaglements per chain (**c**) and the primitive path contour length (**d**) at various values of Wi.

Figure 5 displays the entanglement network properties of the C_1000_H_2002_ melt at various Wi. The ensemble average entanglement density and the probability distribution function for the entanglement density are displayed in Figure 5a,c. Figure 5b shows the tube diameter, determined as the step length of the primitive path $a=\langle L\rangle /\left(\langle Z_{k}\rangle /2\right)$ [2,3]. The probability distribution function of the primitive path contour length is also shown in Figure 5d for various values of Wi. Note that the primitive path contour length, ⟨L⟩, is essentially commensurate with the tube stretch, λ (see Figure 3d), which is the normalized primitive path contour length. These plots show that, within the linear viscoelastic regime, the entanglement network is practically unperturbed as compared to quiescent conditions. Specifically, the entanglement density and tube diameter do not change as the flow strength increases. The probability distribution function for the entanglement density, $P\left(Z_{k}\right)$, follows a Poisson distribution and is independent of Wi in this regime. $P\left(L_{pp}\right)$ exhibits a similar behavior, except that it follows a Gaussian distribution. As Wi increases and shear rate enters into the nonlinear viscoelastic regime, the tube network begins to lose entanglements. Notably, there is no sharp boundary between the second (1<Wi≤38) and the third 58≤Wi<800) flow regimes, as discussed for the structural properties of the system. Rather, there is an initial stage of convective constraint release wherein the chains disentangle at a moderate rate in the region 1<Wi<500 such that $Z_{k}\sim Wi^{-0.07}$. Accordingly, the tube diameter increases moderately in this region. The probability distribution function for the entanglement density, $P\left(Z_{k}\right)$, shifts to the left with increasing Wi as the chains disentangle. The shape of the distribution, however, remains approximately similar to that of the linear viscoelastic regime and still follows a Poisson distribution. On the other hand, $P\left(L_{pp}\right)$ shifts to the right and becomes wider (i.e., with a higher standard deviation); nevertheless, the distribution continues to follow a Gaussian distribution. These results suggest that, although by the end of this flow regime the system loses about 30% of its entanglements, the nature of the entanglement network does not change radically. Note that even at the highest shear rate within this regime, none of the chains has lost all of its entanglements. For instance, the curve for Wi=117 in Figure 5c shows that all molecules possess 5 or more kinks.

At higher flow strength, (i.e., Wi≥585), the entanglement density begins to drop dramatically as Wi increases with a power-law exponent of $\langle Z_{k}\rangle {}^{-0.35}$. The tube diameter also increases substantially in this region, such that at Wi=2340 the tube diameter grows almost as large as the molecular radius of gyration. This means that a molecule could effectively diffuse as far as its size without feeling the confining tube. This interpretation essentially questions the existence of the tube concept and of an entangled system. These subtleties can be understood by examining the probability distribution of the entanglement density. Figure 5c shows that the distributions begin to deviate somewhat from Poisson distributions. More importantly, these distributions suggest that, unlike before, in this flow region some of the molecules have lost all their entanglements and have become virtually unentangled. The distribution of the primitive path contour length also deviates considerably from the Gaussian distribution in this region. All of these observations suggest that the entanglement network is effectively destroyed due to the strong flow. This also might explain why the system behavior at such high shear rates resembles that of a dilute solution, as has been argued for shorter chain C_400_H_802_ and C_700_H_1402_ liquids [3,18]. In this regime, the tumbling cycles are comparatively more regular, similar to those of dilute solutions. Tube stretch approaches its plateau value as macromolecular tumbling becomes the dominant dynamic mechanism.

**Figure 6:**
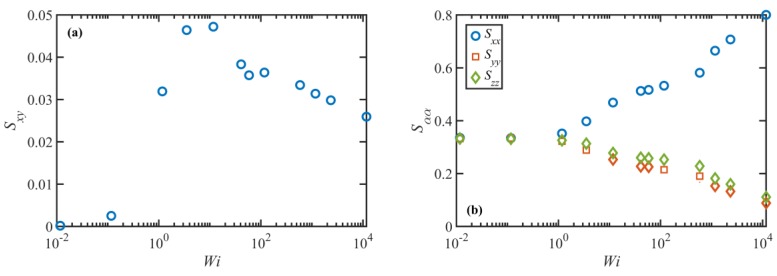
The $S_{xy}$ (**a**) and diagonal (**b**) components of the tube segmental orientation tensor S, as functions of Wi at steady state, as obtained from the NEMD data for the C_1000_H_2002_ melt.

An important characteristic of the entanglement network is the tube orientation tensor, S, that is one of the principal variables of many tube-based constitutive models. Figure 6 displays the nonzero components of S as functions of Wi at steady state obtained from the NEMD data for the C_1000_H_2002_ melt. The average orientation tensor of the tube segments in this figure is defined as $S=\langle u^{t}u^{t}\rangle$, where $u^{t}$ is the unit end-to-end vector of an entanglement strand: knowing the positions of the entanglements (kinks) along the chain from the Z1 code, the end-to-end vectors of the entanglement strands can be easily identified and the appropriate ensemble averages of the components of the orientation tensor can then be readily calculated from the NEMD data. The component, $S_{xy}$, begins to increase at very low Wi within the linear viscoelastic regime. This segmental orientation leads to an increase in the shear stress in this region, in agreement with theory. $S_{xy}$ passes through a maximum in the range 3<Wi<12, which is somewhat higher than the theoretical prediction of Wi~1. At higher shear rates, $S_{xy}$ decreases almost monotonically. Such behavior can lead to excessive shear thinning, as observed in vesrsions of the tube model that do not incorporate CCR, especially within the shear rate range $\tau_{d}^{-1}<\dot{\gamma }<\tau_{R}^{-1}$ or the plateau region where the tube stretch is insignificant. In fact, models that incorporate CCR predict a nearly constant $S_{xy}$, and consequently constant shear stress, in the plateau region in agreement with typical experiments. Hence, the decrease in $S_{xy}$ observed in the NEMD data calls into question the theoretical mechanism of CCR in some tube-based models like MLD. The diagonal components of S remain nearly constant in the linear viscoelastic regime and then diverge from their equilibrium value (~0.33) as Wi increases. At very high shear rates, i.e., $\dot{\gamma }>\tau_{e}^{-1}$, the rate of change in these components increases significantly. This is the shear rate range wherein the entanglement network begins to disintegrate. Generally, the features of these plots are qualitatively similar to those of λ and $\left\langle Z_{k}\right\rangle$ (see Figure 3d and Figure 5a), which could be indicative of an inherent connection between λ and $\left\langle Z_{k}\right\rangle$ with the normal components of the tube segmental orientation tensor. This is specifically important from a modeling perspective as it suggests that the evolution equations for the tube stretch and entanglement density should be expressed in terms of the diagonal components of S rather than the shear component.

**Figure 7:**
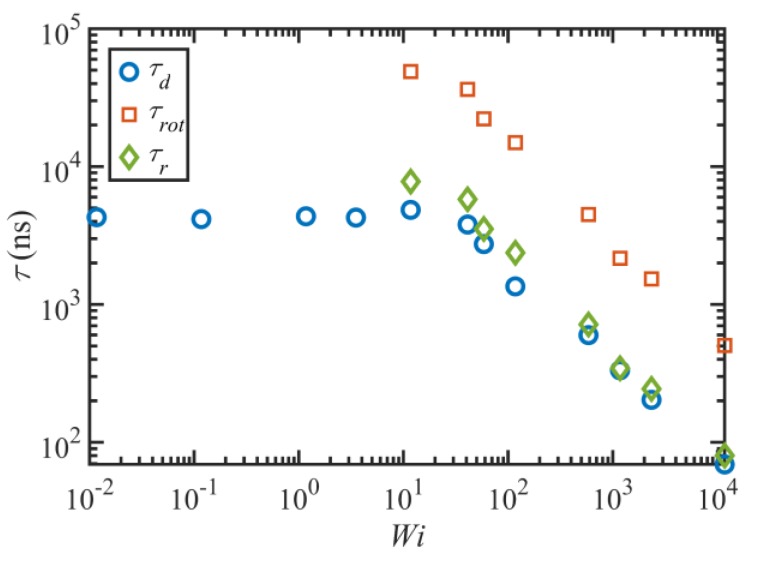
The three characteristic times ($\tau_{d}$, $\tau_{rot}$, and $\tau_{r}$) of the C_1000_H_2002_ melt versus Wi.

Figure 7 displays the important characteristic timescales of the C_1000_H_2002_ liquid as functions of Wi. These timescales are calculated based on fitting the autocorrelation function data of the end-to-end vector with the functional form $\langle u_{i}\left(\tau \right)\cdot u_{i}\left(\tau +t)\right\rangle =\exp\left(-t/\tau_{d}\right)\cos\left(2\pi t/\tau_{rot}\right)$. Hence, $\tau_{d}$ is the decorrelation time of the end-to-end vector, which is equal to the longest relaxation time (i.e., the disengagement time under quiescent conditions and within the linear viscoelastic regime). $\tau_{rot}$ quantifies the period of the rotation and retraction cycle of the macromolecules, assuming that the cycles are quasi-periodic. A characteristic time for the tumbling period can be defined conceptually as $\tau_{r}=\tau_{rot}/2\pi$ [2], displayed as diamonds in Figure 7.

Figure 7 indicates that $\tau_{d}$ does not change significantly within not only the linear viscoelastic region (Wi≤1) but also in the nonlinear regime for 1<Wi≤12. At higher shear rates, the longest relaxation time decreases with a power-law exponent of −0.71±0.06; this is consistent with the scaling exponents of the C_400_H_802_ and C_700_H_1402_ liquids at high shear rates [2,3]. Unlike for the C_1000_H_2002_ liquid, the relaxation times of C_400_H_802_ and C_700_H_1402_ decreased with shear rate at all Wi>1, and hence a separate power-law exponent for the $\tau_{d}^{-1}<\dot{\gamma }<\tau_{e}^{-1}$ regime was reported in prior work [2,3,18]. Nevertheless, a separate scaling factor for low Wi looks to be irrelevant here. This is perhaps caused by the higher entanglement density of the C_1000_H_2002_ melt, which possibly delays any meaningful change in the relaxation time until approximately Wi=10 when $\left\langle Z_{k}\right\rangle$ begins to decrease—see Figure 5a.$\;\tau_{rot}$ also exhibits a power-law behavior that scales as ${\dot{\gamma }}^{-0.7\pm 0.07\;}$with flow strength. Although this value is slightly smaller than those of the C_400_H_802_ and C_700_H_1402_ melts (−0.78 and −0.75 respectively), they are all in reasonable agreement within statistical bounds. The ratio $\tau_{rot}/\tau_{d}$ averages about 7.3 over all Wi≥50, which is reasonably close to 2π, similarly to the prior cases [2,3]. This suggests that a single timescale, one associated with the period of the molecular tumbling cycles, is the sole configurational relaxation mechanism of the C_1000_H_2002_ chains for $\dot{\gamma }\geq \tau_{R}^{-1}$.

#### 3.2.2. Rheological Response

**Figure 8:**
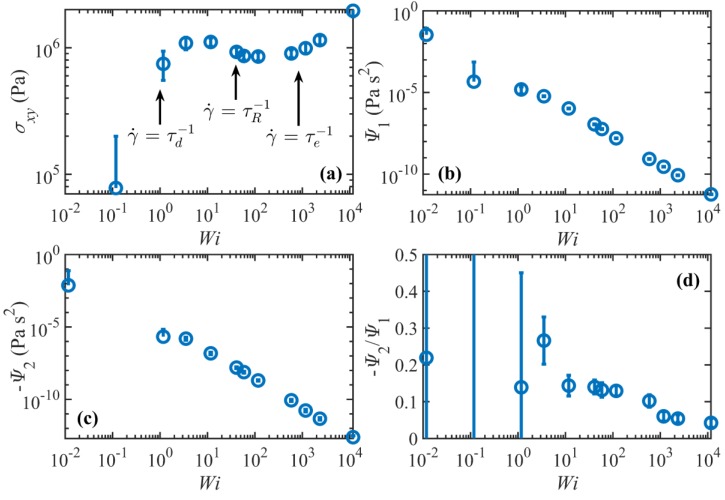
Shear stress (**a**), first and second normal stress coefficients (**b**), (**c**), and the ratio of the normal stress coefficients (**d**), all as functions of Wi.

Figure 8 displays the steady-state rheological properties of the C_1000_H_2002_ liquid as functions of Wi. As expected, the shear stress scales as $\dot{\gamma }$ in the linear viscoelastic regime; however, at higher shear rates, the system’s response is quite different from typical experimental observations, as evident from Figure 8a. Specifically, the shear stress passes through a maximum in the shear rate range 3<Wi<12 and a subsequent minimum in the range 58<Wi<117, in contradiction with the experimentally observed plateau region where the shear stress remains approximately constant or increases slightly as shear rate increases, usually within the shear rate ranges $\tau_{d}^{-1}<\dot{\gamma }<\tau_{R}^{-1}$ or $\tau_{d}^{-1}<\dot{\gamma }<\tau_{e}^{-1}$. Considering the uncertainties of the calculations, it appears that the local maximum and minimum in the shear stress profile occur roughly at about $\dot{\gamma }\sim \tau_{d}^{-1}$ and $\dot{\gamma }\sim \tau_{R}^{-1}$, respectively, and the shear stress surpasses the local maximum value at a shear rate of approximately $\tau_{e}^{-1}$. This possibly implies that the flow is unstable over a fairly wide range of shear rates. Such behavior is enticingly consistent with the discussion of Doi and Edwards (see Figure 7.22 of Reference [34]) concerning the DE model predictions at high shear rates, who argued that the power-law exponent of the shear stress is very sensitive to the relaxation spectrum of the linear relaxation modulus. They argued that the absolute value of the exponent becomes smaller (closer to zero) as the relaxation spectrum becomes broader. Therefore, the shear stress should be approximately independent of the shear rate for polydisperse samples that are commonly used in experiments (hence the plateau), whereas a maximum in the shear stress profile could result from a completely monodisperse sample. Nevertheless, even for monodisperse samples, multiple relaxation processes tend to broaden the relaxation spectrum and weaken the shear rate dependence of the stress. However, as evident from Figure 7, the number of timescales becomes effectively unity for $\dot{\gamma }\geq \tau_{R}^{-1}$. Based on the DE model, $\sigma_{xy}\sim {\dot{\gamma }}^{-0.5}$ for $\tau_{d}^{-1}\ll \dot{\gamma }\ll \tau_{R}^{-1}$ and as $\dot{\gamma }\tau_{R}^{-1}$ becomes close to unity, the shear stress increases due to tube stretching [34]. This implies that if the number of entanglements is not large enough (i.e., $\tau_{d}/\tau_{R}$ is not high enough), the shear rate dependence weakens. In Figure 8a, $\sigma_{xy}\sim {\dot{\gamma }}^{-0.2}$ for 12<Wi<58, which is consistent with this argument.

The plateau region in the shear stress profile has also been postulated to result from the onset of the molecular tumbling cycles that begin to manifest in this shear rate regime [2,3]. This hypothesis led to further investigations which indicated the possibility that shear banding, caused by the molecular periodicity, was a possible cause of the experimentally observed plateau in the shear stress profile [36,37,38]; however, it is unlikely that shear banding occurs in the present simulations since the p-SLLOD equations of motion impose a homogeneous linear velocity profile throughout the simulation cell in the NEMD simulations. That being said, however, recent DPD simulations have demonstrated shear banding in monodisperse polymers in the same range of molecular weight where the flow curve is non-monotonic [36,37,38,39].

For $\dot{\gamma }>\tau_{e}^{-1}$, the shear stress scales as ${\dot{\gamma }}^{0.3}$. The power-law exponents for the C_400_H_802_ and C_700_H_1402_ melts over the same range of shear rates are approximately −0.5 and −0.4, respectively [2,3], which suggest a molecular weight dependence for the shear stress at these high shear rates. Figure 8b,c show the first normal stress coefficient $\Psi_{1}=N_{1}/{\dot{\gamma }}^{2}$, and the second normal stress coefficient $\Psi_{2}=N_{2}/{\dot{\gamma }}^{2}$, where $N_{1}=\sigma_{xx}-\sigma_{yy}$ and $N_{2}=\sigma_{yy}-\sigma_{zz}$. Both coefficients exhibit strong shear thinning behavior in the nonlinear regime with power-law exponents of –1.7 and –1.8, in agreement with those of the C_700_H_1402_ melt [2]. The ratio $-\Psi_{2}/\Psi_{1}$ ranges over $0.04<-\Psi_{2}/\Psi_{1}<0.27$ in the nonlinear regime, again in reasonable agreement with C_700_H_1402_ melt [2] and typical experimental values [40].

### 3.3. Startup of Shear Flow Properties

#### 3.3.1. Transient Behavior

**Figure 9:**
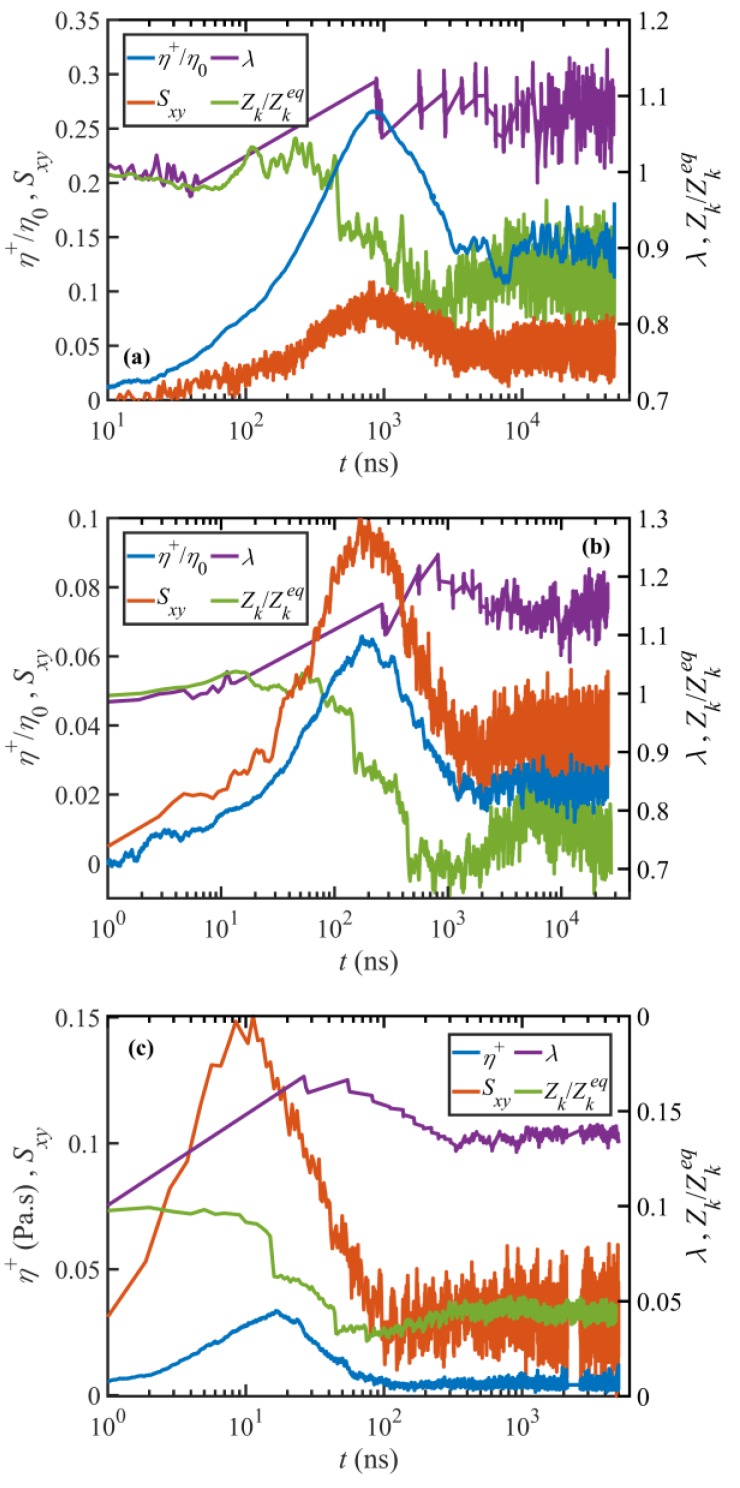
Shear viscosity (blue), the $S_{xy}$ component of the tube segmental orientation tensor (red), tube stretch *λ* (purple), and normalized entanglement density (green) versus time upon startup of shear flow at Wi=12 (**a**), Wi=58 (**b**), and Wi=1170 (**c**). The dynamics of $\left\langle Z_{k}\right\rangle$ are similar to those of λ except that the minimum in $\left\langle Z_{k}\right\rangle$ corresponds to a maximum in λ. Note that in panel (**c**) there appear small gaps in some of the data profiles at long times where simulation data was accidentally deleted. Since these data points had no bearing on the present discussion, the simulations were not repeated.

The time-dependent microstructural and rheological properties of the C_1000_H_2002_ melt were investigated under startup of simple shear flow similarly to those of the C_700_H_1402_ liquid presented in a prior publication [17]. Figure 9 displays the transient shear viscosity, $S_{xy}$, λ, and $\left\langle Z_{k}\right\rangle$ as functions of time for various Wi obtained from the NEMD simulations. The data for the transient viscosity and $S_{xy}$ have been smoothed using a running time average over a number of successive sample times spanning 0.05–0.1 relaxation time at the corresponding Wi, as represented by the circles in Figure 7. It should be noted that λ is very sensitive to the box shape when calculated using the Z1 code; since the box shape continuously changes during the simulation due to the Lagrangian rhomboid periodic boundary conditions, it is difficult to calculate transient tube stretch using the Z1 code. A solution to this problem is to calculate the tube stretch only at time steps when the box is rectangular or slightly (e.g., less than 5%) tilted. The tube stretch profiles displayed in Figure 9 were obtained using this method. A major disadvantage of this method is that it significantly reduces the resolution of data, which could lead to the loss of important dynamical features, such as an overshoot or undershoot. However, unlike tube stretch, the entanglement density is not very sensitive to the simulation box shape; since the entanglement density has essentially similar dynamics as the primitive path contour length (and equivalently, the tube stretch—see Figure 9), it can be used to estimate the overshoot and undershoot times of the tube stretch. Note that an overshoot in tube stretch corresponds an undershoot in entanglement density, and vice-versa.

**Figure 10:**
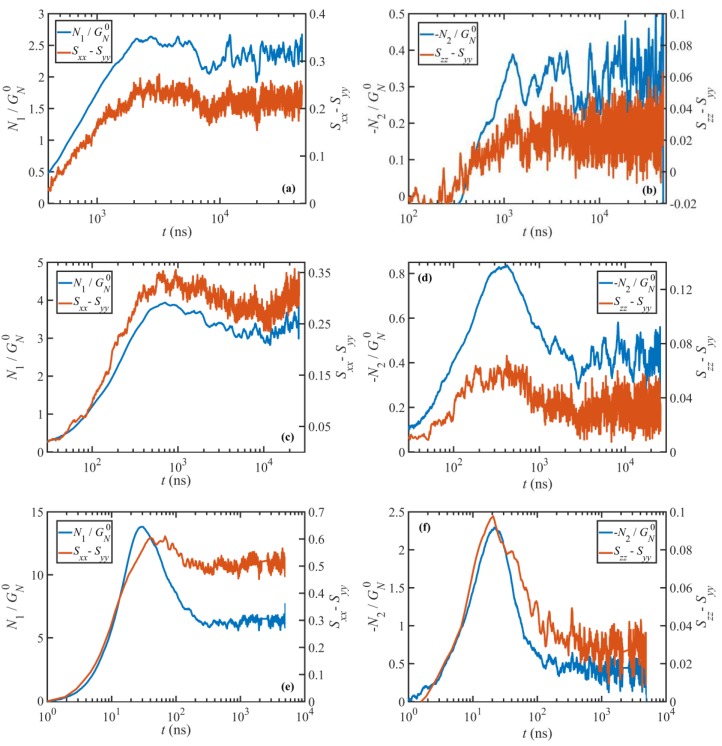
Transient first (left panels) and second (right panels) normal stress differences as well as their corresponding tube orientation tensor differences as functions of time upon startup of shear flow. Normal stress differences are normalized with respect to the plateau modulus. Weissenberg numbers are 12, 58, and 1170 from top to bottom rows, respectively.

The transient first and second normal stress differences are shown in Figure 10 for various Wi as functions of time. This figure also displays the tube orientation tensor differences $S_{xx}-S_{yy}$ corresponding to $N_{1}$ and $S_{zz}-S_{yy}$ corresponding to $-N_{2}$ for comparison. Note that the data have been smoothed using the same method as discussed above. The data are displayed at three Wi numbers, which were chosen to represent the three distinct nonlinear viscoelastic flow regimes: $\tau_{d}^{-1}\leq \dot{\gamma }<\tau_{R}^{-1}$, $\tau_{R}^{-1}\leq \dot{\gamma }<\tau_{e}^{-1}$, and $\dot{\gamma }\geq \tau_{e}^{-1}$.

It is evident from Figure 9 and Figure 10 that the transient viscosity and normal stresses are in qualitative agreement with typical experimental data. Specifically, except for $N_{2}$ at Wi=12, they all exhibit an overshoot for Wi≥12 before they attain steady state. Additionally, the overshoot in shear viscosity is followed by an undershoot at least for $Wi\geq \tau_{R}^{-1}$, again in agreement with typical experiments. These overshoots and undershoots (if any) also occur in the entanglement network variables, as shown in Figure 9 and Figure 10. These figures make it possible to compare the dynamics of the stress tensor with those of tube variables (i.e., the tube segmental orientation tensor S, and the tube stretch λ) to investigate the origins of these phenomena, as discussed in the next section. It is worth mentioning that steady or transient shear banding might occur in the range $\tau_{d}^{-1}\leq \dot{\gamma }<\tau_{e}^{-1}$. This phenomenon cannot be investigated here due to the use of p-SLLOD equations of motion, as discussed in Section 3.2.2. As a consequence, the quantities presented in this section could be affected, assuming shear banding occurs. However, we do not expect a significant change, especially in ensemble-averaged quantities such as the stress tensor and tube variables. Cao and Likhtman [41] compared the startup shear behavior of entangled melts obtained from NEMD simulations using the SLLOD equations and a Langevin thermostat with those of boundary-driven DPD simulations. These comparisons suggested that the ensemble average shear stresses obtained from these two methods were consistent (although not identical) despite the presence of shear banding at the examined shear rates.

#### 3.3.2. Stress Overshoot and Undershoot

From Figure 9, it appears that the dynamic response of shear viscosity and $S_{xy}$ are roughly synchronized over a wide range of Wi. Specifically, the overshoot and undershoot of transient viscosity (if any) occur approximately at the same time as those of the $S_{xy}$ component of the tube orientation tensor. On the contrary, tube stretch and entanglement density respond to the applied flow field with a notable lag as compared to $\eta^{+}$ and $S_{xy}$. It is worth noting that the displayed Wi numbers represent various flow regimes: Wi=12 lies in $Wi_{R}<1$ regime where tube stretch is negligible; Wi=58 is within the regime where tube stretch is significant, and Wi=1170 is within the regime where molecular tumbling is dominant. It should also be noted that this classification is based on the steady-state responses and that it might not necessarily remain valid in transient situations. For instance, whereas the tube stretch is minor at $Wi_{R}<1$, it could exhibit an overshoot in transient situations. Although the magnitude of the shear viscosity (and stress) is a function of both tube orientation, $S_{xy}$, and stretch, λ, these plots suggest that the dynamics of shear viscosity are mainly influenced by the tube segment orientation, $S_{xy}$, which indicates that the principal origin of stress overshoot and undershoot is possibly tube segmental orientation. These plots also show that there is no significant undershoot in λ (or equivalently, an overshoot in $\left\langle Z_{k}\right\rangle$) at any Wi. This observation, that also applies to other shear rates (not shown in Figure 9), practically rules out tube stretch as the origin of the stress undershoot at high shear rates. It is also evident from Figure 10 that the dynamics of $N_{1}$ and $N_{2}$ are also in good agreement with their corresponding components of the tube orientation tensor, i.e., $S_{xx}-S_{yy}$ and $S_{yy}-S_{zz}$, respectively, suggesting that the overshoot in normal stresses arises from the tube segment orientation.

**Figure 11:**
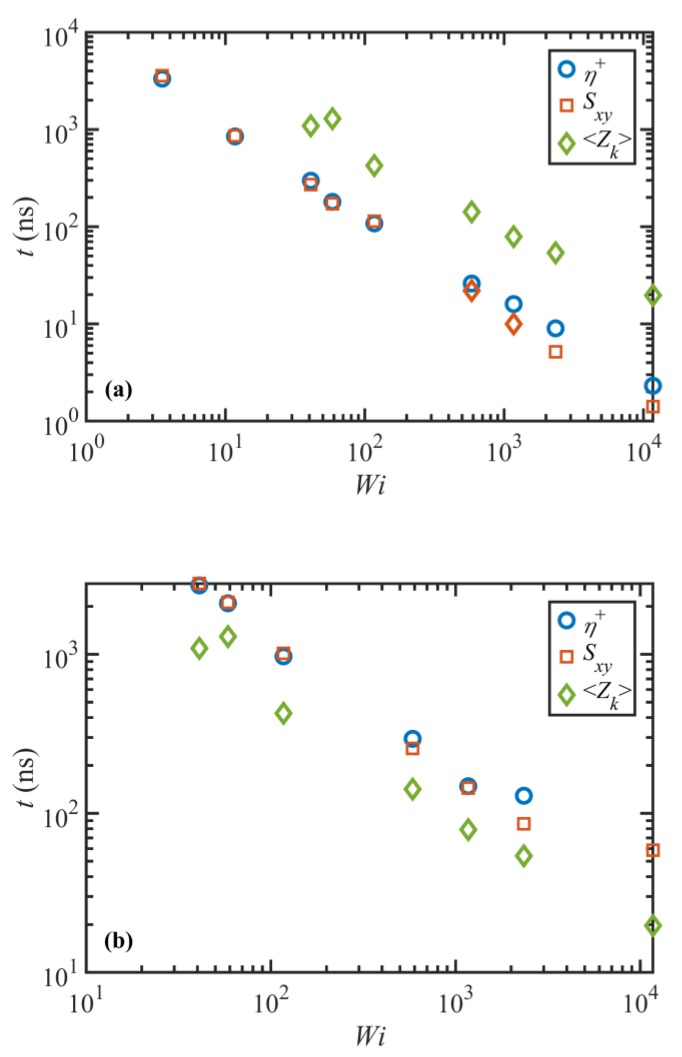
Overshoot (**a**) and undershoot (**b**) times for the transient viscosity, $\eta^{+}$, and $S_{xy}$ component of the tube orientation tensor, as well as the undershoot time for the entanglement density as a function of Wi in both panels. Note that the undershoot time in $\left\langle Z_{k}\right\rangle$ (which corresponds to the overshoot time in tube stretch) does not coincide with either the overshoot or undershoot time in $\eta^{+}$ or $S_{xy}$.

Figure 11 shows the overshoot (panel (a)) and undershoot (panel (b)) times for the transient viscosity and $S_{xy}$ component of the tube orientation tensor as functions of Wi. It also displays the undershoot time for the entanglement density in both panels for comparison. Note that an undershoot in $\left\langle Z_{k}\right\rangle$ corresponds to an overshoot in λ, as discussed before. It is evident that the transient viscosity overshoot and undershoot times effectively overlap with those of $S_{xy}$ at all Wi<585. At higher Wi, although these two curves look to be diverging, the difference between the two times is not significant, considering the error associated with extracting these small values from the noisy data, as shown in Figure 9. On the other hand, it is obvious that there is a significant difference between the undershoot time in $\left\langle Z_{k}\right\rangle$ and either the overshoot and undershoot times of shear viscosity. These results again imply that both the stress overshoot and undershoot are originated from similar phenomena in the tube segmental orientation. This conclusion is in agreement with observations for a C_700_H_1402_ melt at high shear rates [17]. It also agrees with the results of Cao and Likhtman [42] for unentangled and mildly entangled systems, indicating that the origin of the stress overshoot at low shear rates is the orientation of the tube network rather than chain stretching. Jeong, et al. [9] also attributed the stress overshoot to the segmental orientation in a wide range of flow strength for a mildly entangled C_400_H_802_ polyethylene melt. However, unlike the current results, they did not observe a clear overshoot in the primitive path of the contour length (and hence in the tube stretch) even at very strong flow fields. This may be due to the relatively low entanglement density of the C_400_H_802_ molecules used in their NEMD simulations. Masubuchi et al. [43] also investigated the origin of the stress undershoot at high shear rates using primitive chain network simulations. They examined segmental orientation, tube stretch, and the ensemble average squared sine of the chain end-to-end orientation angle (representing the tumbling motion) and showed that all these variables exhibit undershoots, although not synchronized with shear stress. Masubuchi et al. [43] concluded that their results supported the mechanism proposed by Costanzo et al. [16]

**Figure 12:**
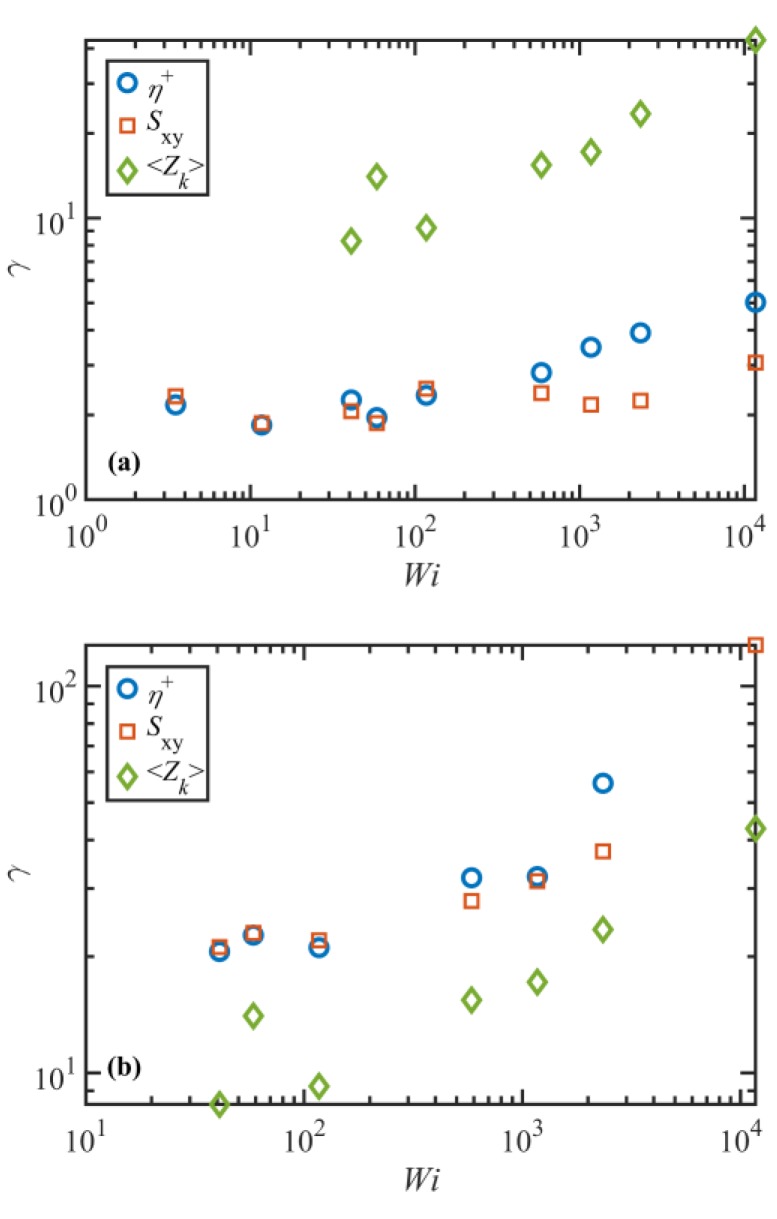
Shear strain, γ, at the times of overshoot (**a**) and undershoot (**b**) in the transient viscosity, $\eta^{+}$, and $S_{xy}$ component of the tube orientation tensor, as well as strain at the undershoot time for the entanglement density as a function of Wi in both panels. Note that the undershoot time in $\left\langle Z_{k}\right\rangle$ does not coincide with either the overshoot or undershoot strain in $\eta^{+}$ or $S_{xy}$. The strain overshoots for $\eta^{+}$ and $\left\langle Z_{k}\right\rangle$ scale as $Wi^{0.17}$ and $Wi^{0.33}$ respectively, for Wi>58.

Figure 12 displays the shear strain, λ, at the times of overshoot (panel a) and undershoot (panel b) in the transient viscosity and $S_{xy}$ component of the tube orientation tensor, as functions of Wi. Shear strain expresses the theoretical deformation due to the applied flow field, which is calculated as $\gamma =\dot{\gamma }t$. This figure also shows the strain at the undershoot time for the entanglement density in both panels for comparison. The agreement between $\eta^{+}$ and $S_{xy}$ curves in the region Wi<585 is not surprising, considering the results of Figure 11 and how the strain is calculated. The important point to notice is that up to very high Wi the overshoot in $S_{xy}$ occurs at about γ=2 consistently. This suggests that regardless of flow strength, the material deforms affinely during the initial 2 strain units until $S_{xy}$ attains a maximum. However, it does not look to be the case at later times that $S_{xy}$ passes beyond its minimum, especially at intermediate and high Wi as evident from Figure 12b. It is also worth noting that the strain at the maximum of viscosity is roughly 2 at low and intermediate Wi, in agreement with the prediction of the Doi-Edwards model [44], and shifts to higher values for Wi≥585. The experimental value of strain at stress overshoot is also about 2 at low shear rates; however, it shifts to higher strains, as the shear rate exceeds $\tau_{R}^{-1}$ [45].

**Figure 13:**
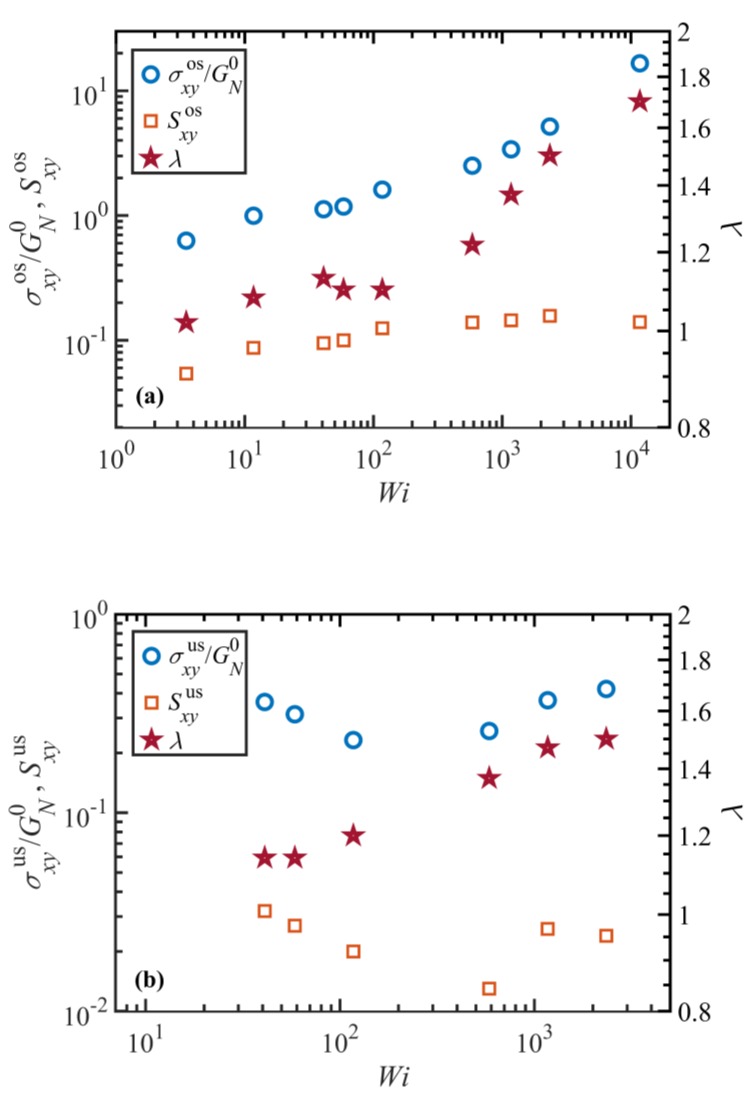
Magnitudes of the normalized shear stress, $\sigma_{xy}/G_{N}^{0}$, the $S_{xy}$ component of the tube orientation tensor, and the tube stretch λ at the stress overshoot (**a**) and undershoot (**b**) times versus Wi. The stress overshoot scales as $Wi^{0.3}$ in the range 3.5≤Wi≤2340, and the tube stretch at the stress overshoot time scales as $Wi^{0.1}$ for Wi>58.

The discussion concerning the overshoot and undershoot dynamics in the last few paragraphs should not lead to misinterpretation about the role of tube stretch in the stress overshoot and undershoot. Figure 13a shows the magnitude of the overshoots in the normalized shear stress and tube orientation $S_{xy}^{os}$, versus Wi. The shear stress is normalized with the plateau modulus, $G_{N}^{0}$. Figure 13 also displays the magnitude of the tube stretch at the time of stress overshoot. Note that this quantity is different from the magnitude of the tube stretch overshoot. It is evident from this figure that for Wi<585 the shear stress closely mimics $S_{xy}^{os}$, while the tube stretch is fairly close to the equilibrium value of unity, or only mildly greater. This suggests that in this region tube stretch has a minor or negligible contribution to the stress overshoot, $\sigma_{xy}^{os}$. At higher shear rates, whereas $S_{xy}^{os}$ looks to become saturated and remain roughly constant, $\sigma_{xy}^{os}$ increases quickly as Wi increases. The tube stretch in this region also begins to increase and diverge from its equilibrium value. This indicates that although the dynamics of the stress overshoot are essentially controlled by the tube segmental orientation (as discussed in the preceding paragraph), its magnitude is significantly influenced by the tube stretch at high flow strength. A similar argument can be made for the stress undershoot—see Figure 13b. This conclusion can be rationalized by hypothesizing that the tube stretch is itself originated from the tube orientation or another dynamic variable. It is however immediately evident from Figure 9 that the $S_{xy}$ component could not be that variable, considering the significant differences between the features of $S_{xy}$ and λ plots in this figure.

**Figure 14:**
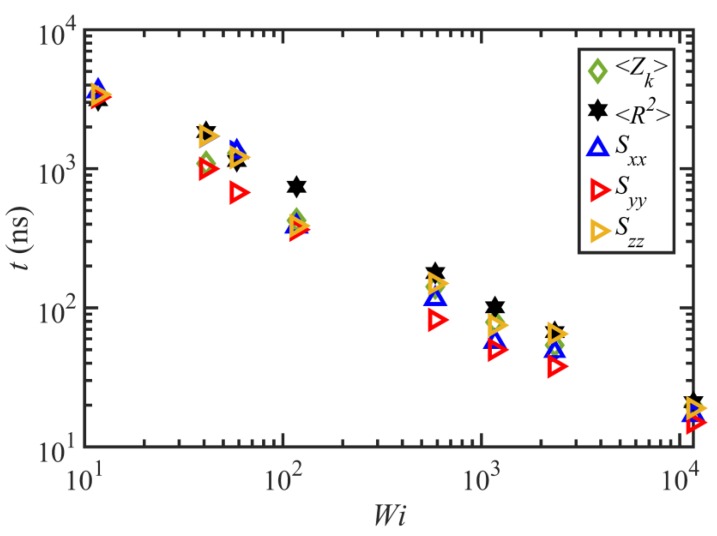
Comparison of the undershoot time for the entanglement density with the overshoot times for the ensemble average squared end-to-end distance, $\left\langle R^{2}\right\rangle$, and the normal (diagonal) components, $S_{xx}$, $S_{yy}$, and $S_{zz}$, of the tube orientation tensor.

Figure 14 shows the undershoot time for the entanglement density as well as the overshoot time for the ensemble average squared end-to-end distance, $\left\langle R^{2}\right\rangle$, and the normal (diagonal) components, $S_{xx}$, $S_{yy}$, and $S_{zz}$, of the tube orientation tensor. Overall, this figure shows that the overshoot times of these variables roughly overlap, within a wide range of Wi including mildly to strongly nonlinear viscoelastic flow regimes. Specifically, there is a good agreement between the undershoot time of $\left\langle Z_{k}\right\rangle$ and that of $\left\langle R^{2}\right\rangle$. It should be emphasized that $\left\langle R^{2}\right\rangle$ is essentially the trace of the ensemble average chain conformation tensor and represents the overall average extensional state of the molecules. Figure 14 implies that the entanglement network, and hence tube stretch dynamics, are mainly influenced by the diagonal components of the orientation tensor or by the overall extensional properties of the molecules rather than the shear component.

## 4. Conclusions

Transient and steady-state dynamic responses of an entangled C_1000_H_2002_ polyethylene melt were examined via virtual experimentation using NEMD simulations. Under quiescent conditions, reptation theory could explain equilibrium properties fairly well. Under steady shear flow conditions, four flow regimes were recognized in agreement with prior results for moderately and mildly entangled C_700_H_1402_ and C_400_H_802_ liquids [2,3]. The first regime was the linear viscoelastic regime ($\dot{\gamma }<\tau_{d}^{-1}$) where most of the structural and topological properties of the system remain unperturbed compared to the quiescent conditions. Orientation effects dominated the rheological response in this flow regime, although they are quite weak. In the second regime ($\tau_{d}^{-1}<\dot{\gamma }<\tau_{R}^{-1}$), the molecules began to align with the flow direction and a significant degree of chain orientation was observed as Wi increased. Additionally, the tube segments began to stretch mildly and chain molecules partially unraveled and disentagled as flow strength increased. However, the dominant relaxation mechanism in this region was the orientation of the tube segments. In the third regime ($\tau_{R}^{-1}<\dot{\gamma }<\tau_{e}^{-1}$), while on average the chains were fully aligned with the flow direction, the molecular disentangling continued and tube stretching dominated the rheological response. Additionally, the rotation of molecules became a significant source of the overall system dynamics. In the fourth regime ($\dot{\gamma }>\tau_{e}^{-1}$), the chain stretching decelerated, and tube stretch approached a plateau value. At the same time, flow-induced disentanglement continued and the entanglement network began to deteriorate such that some molecules became completely devoid of entanglements. The molecular tumbling, on the other hand, gradually became the dominant relaxation mechanism, and molecular configurations followed more regular cycles when compared to similar behavior at lower flow strength.

The comparison of transient shear viscosity, $\eta^{+}$, with the dynamic responses of key variables of the tube model, including the tube segmental orientation, S, and tube stretch, λ, revealed that the stress overshoot and undershoot in steady shear flow of entangled liquids were essentially originated and dynamically controlled by the $S_{xy}$ component of the tube orientation tensor, rather than the tube stretch λ, over a wide range of flow strengths (including shear rates faster than $\tau_{R}^{-1}$). Nevertheless, the magnitude of the stress is significantly affected by λ at high shear rates.

## Figures and Tables

**Table 1:** Details of the simulation cells: $L_{x}$, $L_{y}$, and $L_{z}$ are box lengths in the x, y, and z dimensions, respectively.

Wi	$L_{x}(Å)$	$L_{y}\;\mathrm{and}\;L_{z}(Å)$	Number of Particles
0–1.2	84.7	84.7	20,000
3.5–12	169.3	84.7	40,000
41–58	254.0	84.7	60,000
117–11,700	508.0	84.7	120,000

**Table 2:** The three relaxation times for the C_1000_H_2002_ melt calculated according to reptation theory and segmental mean-square displacement (MSD) data.

Relaxation Time	Theory	MSD
$\tau_{e}$ (ns)	6.4	2.7
$\tau_{R}$ (ns)	137	194
$\tau_{d}$ (ns)	5305	5834
